# Crisis Work Embedded in a Global Crisis: The Early Phase Impact of COVID-19 on Survivors of Intimate Partner Violence and Service Provisions

**DOI:** 10.3390/ijerph19084728

**Published:** 2022-04-14

**Authors:** Vithya Murugan, Terri L. Weaver, Theresa Schafer, Quin Rich

**Affiliations:** 1 School of Social Work, Saint Louis University, St. Louis, MO 63103, USA; tschafer301@gmail.com (T.S.); quin.rich@slu.edu (Q.R.); 2 Department of Psychology, Saint Louis University, St. Louis, MO 63103, USA; terri.weaver@health.slu.edu

**Keywords:** COVID-19, intimate partner violence, service providers, resiliency

## Abstract

COVID-19, as a global pandemic, was a public health inflection point for individuals affected by intimate partner violence (IPV) and those who provide IPV services. Public health guidelines that were intended to reduce risk of exposure to the virus impacted vulnerability factors for IPV survivors and associated systems of services. We aimed to (1) explore the effect of COVID-19 on survivors of IPV; (2) assess the effect of COVID-19 on IPV-related service provisions and service providers; and (3) explore challenges and opportunities in the wake of COVID-19 on broader IPV services and advocacy. Method: Twelve directors of IPV shelter, criminal justice, and other advocacy services within a diverse, Midwestern metropolitan area were recruited to participate in in-depth, semi-structured interviews in June–August 2020. Interviews were transcribed verbatim and analyzed using Dedoose. Data were coded and analyzed through thematic analysis. Results: Four major themes, contextualized by COVID-19 and racial injustice, emerged from the data analysis: (1) IPV-related trends; (2) impact on IPV survivors, services, and agency morale; (3) inter-agency collaborations; and (4) future opportunities for innovative service delivery. Gaps and opportunities for developing culturally congruent, trauma-informed services were identified. Conclusion: Findings suggest that responsive and accessible IPV resources and associated advocacy services can make the difference between life and death for survivors.

## 1. Introduction

Public health officials asserted in March 2020 that it is safest to be at home to reduce risk of exposure and to curb the spread of COVID-19 virus. However, professionals who work with survivors know the home can magnify risk of exposure to another public health hazard: intimate partner violence (IPV), including emotional, physical, psychological, and sexual violence. Individual and relational level stressors exacerbated by the pandemic, including anxiety and fear related to unemployment, sickness, and death, may increase the likelihood of conflict and the use of maladaptive coping mechanisms, such as alcohol and substance use [1]. These stressors are nested within structural contexts that include chronic and persistent poverty, which may exacerbate the frequency and severity of IPV [2,3]. Self-isolation forced those affected by IPV and their children to be confined, in proximity, with their abusive partners with no definite endpoint. Additionally, early public health recommendations, such as social distancing, limited individuals’ options for access to IPV agencies and associated resources—at least in their conventional form. Research has confirmed that COVID-19 lockdowns have led to decreased service utilization by IPV survivors, increased severity of IPV, and experiences of isolation [4,5,6,7,8,9,10]. Concerted efforts by IPV service providers to effectively respond to the COVID-19 pandemic have been identified as crucial for positive survivor outcomes [11]. However, perspectives from IPV service providers on the early stage of the pandemic have received limited scholarly attention.

Previous quantitative research conducted in the same Midwestern city and over the same time as the present study identified survivor concerns with decreased safety, loss of access to support services, increased economic precarity, and forced contact with perpetrators as key concerns [12]. According to service providers, the isolation of the pandemic highlighted particularly acute elements of intimate partner violence, including substance use coercion, reproductive coercion, and control around heath care and service seeking [13]. While the pandemic has increased vicarious trauma and exacerbated pre-existing barriers for culturally specific agencies, service providers have responded creatively, such as utilizing technology to connect with survivors [14]. In line with findings from the present study, Black survivors have been particularly affected by pandemic barriers to accessing IPV services. Data from Chicago, a nearby city, indicated that IPV-related police reports from the majority of Black neighborhoods decreased at almost twice the rate of the general population due to stay-at-home orders [15].

The present study builds on the findings of previous studies by exploring service providers’ perspectives on the specific factors that promote or inhibit survivor safety in greater depth. As articulated by Ragavan and colleagues [13], service providers can share information about how COVID-19 has affected survivors without causing safety concerns intrinsic to data collection with survivors during a global pandemic. As such, we aimed to (1) explore the effect of COVID-19 and associated public health guidelines on individuals affected by IPV; (2) assess the effect of COVID-19 on service provisions for individuals; and (3) explore challenges and opportunities in the wake of COVID-19 on IPV services and advocacy.

## 2. Materials and Methods

### 2.1. Participants

Participants were recruited through convenience sampling via the End Domestic Violence Network (EDVN) listserv consisting of over 20 member agencies in the Midwest metropolitan area. To participate in the study, participants needed to be 18 years or older and needed to have worked at an IPV agency in the Midwest metropolitan area as an executive director, program director, and/or advocate. Interested participants were asked to phone or email the researcher, who then provided them with additional information about the study. If the participant indicated that they were interested, an interview date and time was arranged. Interviews took place over Zoom and ranged in duration from 60 to 90 min. Participants were compensated with a USD 20 Amazon gift card. A total of 12 participants were interviewed by the first and/or second authors, both of whom are doctoral-level researchers trained in qualitative methods. The sample of 12 women was sufficient for the analysis because saturation was achieved such that no new data or theoretical insights continued to emerge [16]. The majority of participants were women (92%, *n* = 11). Their ages ranged from 25 to 70 years with a mean of 42.25 years. The participants’ length of time with their respective agencies ranged from 1 to 20 years with a mean of 8.85 years at their current agency and 4.25 years in the current position.

### 2.2. Interview Guide

Semi-structured interviews are used as a method of data collection to gather information from participants who have “personal experiences, attitudes, perceptions, and beliefs related to the topic of interest” [17] (p. 2). The semi-structured interview guide contained approximately 15 questions and addressed the following topics: service providers’ perceptions of how COVID-19 has affected survivors of IPV; how COVID-19 has affected services available to survivors; and what is needed to effectively support similar agencies in helping survivors. Planned and unplanned follow-up probes were utilized to deepen the PIs’ understanding of the service providers’ responses. Planned follow-up questions are questions that ask for more details about particular aspects of the core questions [17]. For example, one question on the interview guide asked how COVID-19 affected survivors of IPV. Planned follow-up questions included: How has it affected severity/frequency of abuse? How has it affected mental and physical heath? How has it affected caregiving of children? Work? How has it influenced the tactics used by batterers? Unplanned follow-up questions were also utilized to seek clarification on words and phrases to avoid assuming shared meaning. For example, the PIs would ask “Could you tell me what that means or looks like for you?” or “Could you give me an example?” to encourage elaboration.

### 2.3. Data Analysis

The interview data were transcribed and analyzed using Dedoose, a cross-platform software often used for qualitative and mixed-methods research. The research team employed a thematic analysis approach, defined as “a method for identifying, analyzing, and reporting patterns within data” [18] (p. 79). Due to its flexible approach, thematic analysis allows for rich, detailed, and complex descriptions of the data.

The first and second authors and a master’s-level research assistant (third author) began by familiarizing themselves with the data by reading each transcript in its entirety. Each documented their thoughts about potential codes which they shared and discussed during a debriefing session. Next, a preliminary codebook, containing codes and their definitions, was developed based on questions from the interview guide and codes that were discussed during the debriefing session. One transcript was then selected for preliminary coding by the first, second, and third authors, ensuring that the initial codebook adequately captured patterns in perspectives across the interview. Coders then met to review and compare their coding, and discrepancies were discussed until a consensus was reached. This led to the first refinement of the codebook which included the addition of new codes, clarification of code definitions, and inclusion of exemplars. The three authors then recoded the initial transcript using the refined codebook.

As the discrepancies between coders were minimal and not code-specific (i.e., the length of excerpt coded), all remaining transcripts were double-coded by the authors. The full team met, as needed, to discuss and resolve any coding discrepancies until minimal discrepancies emerged. The team then met and discussed the consolidation of codes into broader themes. Quotes were extracted from codes for each theme and then re-evaluated to ensure they captured the meaning of themes [18]. Further, to ensure the accuracy and reliability of the qualitative data, member checking was conducted with the participants. Following the completion of analysis, the researchers sent a draft of the report to the study participants. The participants were asked if the findings reflected their experiences and if they wanted to change or add anything. Participants generally confirmed the findings as reported.

## 3. Results

Interviews revealed four, early-phase COVID-19 themes: (1) intimate partner violence (IPV)-related trends; (2) impact on IPV survivors, services, and agency morale; (3) inter-agency collaborations, and (4) moving forward—lessons learned and ongoing needs.

### 3.1. IPV-Related Trends

All participants discussed IPV-related trends since the emergence of COVID-19 and the subsequent implementation of stay-at-home orders. The most palpable trend was the precipitous decrease in hotline and emergency shelter calls. For example, an executive director reflected on the dwindling number of hotline calls that her agency received, “I [also] work our crisis line and our [call] numbers went down…”

The executive director, who oversees emergency housing, shared:

The biggest thing [COVID trend] that I can speak to right now is the decrease in calls [shelter] that we have seen since the pandemic started. When the pandemic was officially declared [a national emergency] in mid-March, we had six families in [the] shelter so we were not full and that is abnormal for us. We typically are always full. And if we have a space, we have like one space [available]. So, to have three [spaces available] …

The executive director shared that she partially attributed the decreased volume in calls to the rapidly evolving news cycle. At the outset of the pandemic, media outlets covered the effects of COVID-19 on domestic violence by interviewing service providers. Information that was presented at one time may have quickly changed or even become obsolete soon thereafter. The executive director stated: “There was a lot of misinformation out there. There was a lot of news articles reporting that shelters were not taking people. And we were [taking people]. So, we did our best to reach out [to the media] every time we’d see those stories…”

Despite the decrease in calls for supportive services, many participants noted an increase in the incidence and severity of abuse reported by the survivors with whom they did have contact. A service provider described her perception of the escalating violence severity:

Now [during the stay-at-home orders] it seems that that abuse is consistent and escalating. I have several women [clients] right now who are in the middle of extremely abusive relationships. Um, I’ve had two women within the last couple of weeks that actually had to go to the hospital for their injuries.

In fact, some participants attributed these trends to survivors’ diminished prioritization of their physical safety during COVID-19. More specifically, participants noted that the pandemic has forced survivors to prioritize other competing demands (e.g., employment, childcare), sometimes at the expense of their physical safety. While these demands are omnipresent in the lives of survivors outside of the COVID-19 context, participants reflected on how the pandemic has caused an inflection of diminished prioritization of safety due to role strain. For example, another service provider shared:

It [survivors’ personal safety] kind of has had to be deprioritized as they’ve [survivors] had to homeschool and had to figure out employment and childcare and all the things that all of us are dealing with in the midst of [the pandemic]… you know being in an abusive relationship and having to potentially live with the abuser. I would say that’s one of the biggest trends we’ve noticed is just sort of the de-prioritization of safety measures that normally, we would very much be prioritizing under normal circumstances if those other, you know, [other] resources were in place

In addition to using stay-at-home orders to further isolate survivors, participants described how perpetrators have used economic abuse strategies to control survivors, including forcing them to work or not work, or to intercept their stimulus checks upon arrival. Another service provider discussed how perpetrators attempt to get money from survivors’ stimulus checks:

When the checks were going out, a number of women that were speaking to me [shared] that their significant other [was] trying to like control… like checking the mailbox and, and [asking] ‘has your check come yet?’ like constantly badgering… to make sure that they kind of got first dibs on the [survivors’] check.

Many participants underscored the importance of clarifying misconceptions that suggested that COVID-19 caused IPV. Instead, participants shared that the pandemic increased the incidence of IPV by exacerbating existing stressors:

COVID did not cause domestic violence. COVID has amplified [IPV] that, and made those pressures even greater, that has led to increase incidence and an increased impact. So, so often people will ask, like, so is COVID causing more domestic violence? No, you need to know the root of domestic violence and that COVID is a layer on top of that, that influences the impact of incidents.

### 3.2. Impact on IPV Survivors, Services, and Agency Morale

#### 3.2.1. Survivors

All participants explicitly discussed the adverse economic effects experienced by survivors that were either caused or exacerbated by the pandemic. Participants shared that largely due to COVID-19, many survivors and their families grappled with job loss or instability, housing insecurity or homelessness, and lack of childcare. For survivors of color, the economic implications of the pandemic have been especially pronounced, largely due to systemic inequality. For example, one participant shared: “Eighty percent of the people we serve are Black. And because they may not have other resources, because they, you know, they’re also living below the poverty line and they didn’t graduate high school and none of that’s a coincidence.” Additionally, participants shared that most of the jobs that have been available to Black survivors during the pandemic have been in essential roles, thus increasing their risk of exposure to COVID-19. Another participant shared:

[Most] of my ladies are African Americans, and we’re finding them jobs in these essential roles. That means they’re going to be overrepresented in those essential worker positions where they’re going to be exposed to COVID-19 at a higher rate, than those of us who could work from home…

Participants reflected on how these economic challenges intensified already precarious relationships and placed survivors at an increased risk of experiencing IPV. For example, a participant explained:

The biggest complaint [from survivors] is that a lot of people [survivors and their families] lost their employment. I think that financial stability does play a part in the stability of the home. And so, now, if you take a situation that may have [already] been volatile, and then now put on top of it financial stress, right, you know, the question, is that at a tipping point [for IPV]?

Many participants shared survivors were acutely aware of the risk that financial insecurity during the pandemic created for them whilst experiencing IPV. Another provider described how a survivor anticipated that her partner’s unemployment and increased alcohol consumption would foster a dangerous dynamic within the home:

She [the survivor] knew the triggers well enough to know he’s [her partner] going to be home, he’s going to be drinking, he’s not going to be getting a paycheck until his unemployment starts, this [the violence] is going to get bad, it’s [the violence] going to get worse.

Furthermore, some participants shared how economic hardships exacerbated by the pandemic coupled with the stay-at-home orders have halted survivors’ plans of leaving abusive relationships. One participant shared how many survivors who considered moving in with family and/or friends were unable to do so because of social distancing guidelines:

So [survivor’s] family and friends, what I heard…were not an option [to stay with] because of social distancing. As they [survivors] were kind of working towards independence, that [staying with family and friends] wasn’t going to be an option because of social distancing.

Additionally, in some instances, participants shared that they returned to their abusive partners during COVID-19 out of financial necessity. Another participant shared:

Folks [survivors] need a car, they need [financial] stability…to be able to be independent, you know, it’s like, the lack of financial stability, often we [service providers] see folks going back to an abusive partner because they’re just not making it [financially]. And I worry about that a lot.

Participants also reflected on the challenges that survivors experienced in accessing support services during the pandemic. Although they shared that their respective agencies were able to quickly pivot certain services (e.g., counseling, protection order filing, safety planning) to virtual platforms, barriers to accessibility persisted for survivors. One barrier that survivors encountered was the lack of privacy in their households (due to stay-at-home orders) that often prevented them from being able to meaningfully participate in counseling and/or safety planning sessions.

Additionally, participants noted that many survivors do not have access to computers in any form and may have limited data on their smart phones, and thus, are unable to take advantage of virtual support services. In fact, some participants noted that this was an issue that was especially pronounced for clients located in a Midwest City. This service provider shared: “A lot of the clientele that I work within the city do not have access to desktop or laptop, laptop computers…we’re trying to figure that out a little bit.” Although participants acknowledged that smartphones and tablets are virtually ubiquitous, they shared that certain services (i.e., court filing systems) are not mobile-friendly and, therefore, may not be accessible to all survivors. Additionally, in some cases of IPV, survivors were limited in their phone use to access services for fear of their abusive partner accessing and tracking their call logs.

Many participants openly reflected on systemic racism and ongoing police brutality against Black men and women. Specifically, participants shared how these insidious social issues serve as barriers to Black survivors calling the police for assistance, even in acute circumstances. This participant explained: “There are in the current climate, there are some victims that are rightfully 100% afraid to seek police assistance… [Black] Victims don’t often call the police because they’re scared…”.

#### 3.2.2. Services

All participants discussed the adaptations that their respective agencies made to IPV services in response to the pandemic. When COVID-19 was declared a national emergency, participants shared that their respective agencies devised and implemented safety protocols to reduce the risk of exposure to the virus. These protocols included limiting the number of in-person staff members; procuring COVID-19 testing and protective personal equipment (PPE); and, where possible, pivoting service offerings to a virtual format.

After barring minor roadblocks that included limited access to stable WiFi and concerns about firewall security, participants shared that their respective agencies made relatively quick transitions to virtual service offerings. Yet, many participants shared that the quantity and quality of the services offered were largely affected. For example, many participants shared that the way their agencies approached outreach with survivors of IPV was affected by the pandemic. Participants shared that their agencies began to rely heavily on social media to reach out to survivors of IPV during the pandemic. A participant shared:

There’s been a continued focus on the use of our social media, to reiterate to folks that we’re still providing services. They [the agency] started up a newsletter, oh, gosh, it was probably about two months ago. And what they [the agency] would do is highlight each of our services and explain what that looks like with COVID.

Participants shared that many services, such as intake, safety planning, and counseling, were significantly altered in their virtual transition. All participants shared that the lack of in-person, tactile interactions with their clients adversely affected the nature of services being offered, specifically related to rapport building with clients. Additionally, participants relayed that the lack of privacy in survivors’ homes often inhibited their ability to provide quality services to their clients. Another provider described the complexity of offering virtual counseling services to clients, especially when they were cohabitating with their abusive partner:

With phone appointments, sometimes that person [abusive partner] is there… And so, it’s kind of a situation where we [survivor and counselor] must text in advance to see if they’re [abusive partner] there. And then, you know, sometimes we’re [survivor and counselor] on the phone and then that person [abusive partner] comes home and then [the survivor says] ‘Okay, we got to get off the phone real quick.’ Whereas, you know, in the past, like I said, you know, [survivor and counselor are] meeting like at a library or something like that, we just never had to worry about that kind of stuff, you know.

Participants also reflected on services that were difficult to translate to a virtual platform, including clinical therapeutic interventions used to treat trauma or play therapy with children. This participant shared the challenges of virtually administering eye movement desensitization and reprocessing (EMDR):

Our counseling services, as they still are currently all either virtual through telehealth or they’re on the phone. What’s kind of tough about that is in talks with counselor friends that I have it’s [counseling] very different and so there may be certain techniques such as EMDR [eye movement desensitization and reprocessing], which is a form of really beneficial [therapy] for trauma victims… you can do it [EMDR] virtually, in some cases, but it’s like a really heavy form of therapy that we really don’t want to do with somebody over the phone or over video calls. So I know that’s been impacted.

The participant continued by describing the challenges associated with providing therapy to children and the adaptations that her agency made to accommodate children’s needs. The participant shared:

[The agency’s] Our child therapists… I’ve talked to multiple of them, I’ve talked about how realizing that kids’ attention spans virtually is very different [than in] their [face-to-face] life. So we’ve gone from hour [therapy] sessions to like 30 min because kids don’t have that attention span to look at a screen and do therapy.

Participants shared that some services, especially those offered in group formats (e.g., group therapy, children’s support groups), had been suspended due to social distancing guidelines. A participant expressed her fear for children exposed to IPV who may not be getting the communal, peer-based support that they were receiving before COVID-19:

It’s terrifying to think of children who aren’t in shelter because we were so much more than just a roof over their head… We had community nights, we had art therapy, we had support dogs and we really had a great program. And just knowing that there’s so many kiddos that are stuck and not being in school and not having sort of all those normal mechanisms, and that are stuck in homes.

The effect of COVID-19 on IPV service provisions was especially palpable for under-resourced agencies that predominantly worked with ethnic minority survivors and/or survivors living in a Midwest City. When it came to legal and court services continuing through the pandemic, the economic disparities between courts in certain geographic jurisdictions became acutely highlighted. Because the Midwest County has a specific court dedicated to domestic-violence-related issues, participants shared that the county courts were able to renegotiate court services and access virtually more easily and quickly, whereas city courts struggled to shift with the same ease. Specifically, with regards to filing orders of protection, the county adopted an online filing system early in the pandemic, while the city was slower to adopt these measures.

Participants shared that survivors’ IPV-related needs are unique and often require services that are not typically housed within a traditional IPV agency (e.g., substance abuse treatment and acute mental health services). Unfortunately, participants noted that many agencies providing such services were forced to suspend services or close due to COVID-19, thus leaving gaps in service provisions for survivors. Another participant explained:

With COVID, a lot of those [allied services] agencies are folding up. It’s, it’s you know, frustrating that, you know, they just closed up and there’s no real safety net to kind of, to continue to provide some of these services [for survivors], particularly, like I said, some of the drug treatment ones, those are the ones that I’m, I’m pretty concerned about right now.

#### 3.2.3. Agency Morale

Agency reflections on morale and the response to low morale highlighted the juxtaposition of the crisis worker embedded within a global crisis and the associated constraints on the work that reflects the mission and worker identity. Most participants mentioned the strain on personnel during this time, including increased role strain associated with having family and extended family, childcare and work coalesce at home, as well as the challenges to the mission and identity of domestic violence advocacy with COVID-related restrictions abruptly limiting available space or types of service activities. Reflecting upon her staff, one participant said: “We got a lot of pushback when we had to say, ‘we have to limit the number of intakes,’ or ‘we can’t do intakes for a few days.’ Like they were really pissed because it was like, ‘This is what we do. Women are still at-risk; women are still dying’”.

Effectively coping with threats to morale included transparent communication (what was known and what was not known at the time regarding COVID-19 safety protocols or shifting policies), leaning on one another for support, flexibility, and allowing work-related accommodations. Thus, personal limit-setting and self-care were supported. One provider shared: “I think that, I think that we keep a positive attitude around here. We, like I said, are each other’s support system. We have a great chemistry with each other. And so we feed off of each other”. Another service provider reflected: “I think we just, you know, hear what the needs are and try to honor and help kind of navigate as best as we can”.

Several of the agencies mentioned the importance of tangible supports, including external as well as internal funding that helped to secure employees’ jobs, and at least one agency highlighted the importance of formalizing health-related organizational policies to protect their employees. The service provider from this agency stated: [We found] that if we had a public health leave policy, staff would still get paid in full. So we very quickly developed a policy and got it approved by the board.

Other tangible support included staff group activities that could be completed remotely and shared with the group—often with a focus on health and personal wellness, such as a yoga or walking challenge. Additional morale boosters mentioned by at least one group were tokens of appreciation, such as food or T-shirts.

### 3.3. Inter-Agency Collaborations

Almost all participants discussed agencies that they collaborated with during the pandemic. Yet, many participants noted that the IPV sphere, pandemic or not, seldom operates in isolation, and that inter-agency collaboration is crucial to optimally assisting survivors of IPV. For example, an IPV court advocate explained:

I would say, pre-COVID and post-COVID and under any normal circumstances, the domestic violence [IPV] court does not operate in isolation. I mean, we exist partially because of our community partnerships and collaboration. We [IPV court] consider ourselves very much a part of the community wide efforts. People’s experience with the court system is usually fleeting, you know, they may come to court once and then we never see them again, they may be there more continually. But the reality is we if we have that opportunity to connect with a victim, we need to make sure that we’re connecting them to those long-standing community resources…

All participants represented agencies that were members of the EDVN. When COVID-19 was declared a national emergency, participants shared that an EDVN meeting commenced, which assessed the functionality and capacity of member agencies during the pandemic. A working list was disseminated across EDVN member agencies and updated as the pandemic unfolded.

Some participants shared that they collaborated with agencies outside of EDVN to meet survivors’ needs during the pandemic. For example, one participant shared that her agency formalized a collaboration with the local counseling center to incorporate trauma-informed services

### 3.4. Moving Forward and Lessons Learned

Participants were asked to reflect upon the lessons learned as they navigate the process of providing domestic violence-related services during the pandemic and priorities for moving forward. Most of the participants highlighted the key role of technology and the importance of investing in technological upgrades for the agency, including boosting WiFi capacity and enhancing firewalls, purchasing new computers for staff, and supporting survivors in becoming more technologically savvy to engage in telehealth services. Several agencies mentioned that this focus on technology was a significant shift in budgetary emphasis; thus, agencies had to reframe their perceptions of this investment as integral to connecting survivors with existing services and keeping them safe while doing so. Several agencies also mentioned some of the funding limitations of existing grants with allowable expenses primarily for direct services. Other agencies mentioned emerging grants with a focus on building technological infrastructure. One service provider explained: “We have gone into the technology world kicking and screaming the whole way. And that is only for good reason. Like, you know, but so when something like this hit, we did have to like … we had to buy laptops for everybody”.

At least one agency mentioned the importance of developing or adapting existing technology platforms to enhance access while also reducing risk to the safety of survivors as they communicate remotely. One service provider noted:

…if somebody got a hold of her phone or, you know, are there certain platforms that you can do that will sort of like Snapchat that will instantly delete text or so we started researching it not in connection to this just something in the long term. Would that be part of our technology goal. I wish we would have had it, it would have made a big difference.

## 4. Discussion

Our study sought to explore the early-phase impact of COVID-19 on survivors of IPV and related service provisions. Taken together, the trends highlighted in this study demonstrate how COVID-19 amplified the risk for an exacerbation of IPV by inflated IPV-related relational dynamics, including economic abuse, isolation, and more severe assaults. Additionally, COVID-19 created additional barriers to accessing supportive services, including role strain, thus limiting opportunities for women to focus on their health and well-being and proximity to abusive partners precluding opportunities to reach out to services and to have private conversations. IPV is interwoven with macrosystems [19] and changes within those macrosystems, including pandemic-related environmental stressors, which have a profound impact on both exposure and IPV, particularly for those who are more disadvantaged. These findings also underscored the multiplicity of safety threats during this time, both within and outside the home, and the resulting challenges for safety planning and decisional processes about reducing risk of harm [20]. With the expanded landscape of COVID-19-related risk factors, respondents reported that survivors made fewer overall calls but ultimately reported serving survivors who experienced more severe violence. This increase in severity suggests that there may have been new benchmarks for turning points as survivors struggled to navigate fewer available resources, thus increasing responsibility and the uncertainty associated with the early phase of the virus. Holistically acknowledging the compounding intersection of diseases and the social, environmental, and economic barriers to freedom and healing already faced by IPV survivors is an essential first step in understanding the experience of survivors during the COVID-19 pandemic and could serve as a model for including the influence of macro-systems, including systemic racism, within individualized safety plans.

Specifically, our study revealed the added vulnerability of BIPOC survivors, exacerbated by the intersection of IPV, COVID-19, and ongoing systemic racism [13,19]. Findings also underscore the ways in which some conventional recommendations intended to enhance survivor safety, as advice to ‘call the police if you are in imminent danger’ may not be a viable option for BIPOC survivors or for those who witness IPV happening to BIPOC survivors due to concerns of enhanced risk [21]. These findings serve as a clarion call for IPV resources and services to evaluate the degree to which they are offering culturally congruent services for diverse populations and raise critical questions about whether this is a time for new paradigms for survivor-centered services. IPV services have hardly changed over the past forty years and the voices of minoritized individuals have been largely absent from the design of extant services [22,23,24].

Additionally, our study found that the effects of COVID-19 were especially palpable for under-resourced agencies that predominantly serve BIPOC survivors, underscoring the financial fragility of these systems and the need for increased investment of resources into the formal and informal agencies serving these women. While the overall prevalence of IPV has decreased with the infusion of resources (e.g., Violence Against Women Act (VAWA)), disparities for marked or increased risks of homicide, and the different impacts of preventive policies faced by Black women (compared with other racial groups)) [25]. These findings highlight the eminence of creating intervention strategies that address IPV, service provisions, and COVID-19 within the context of existing systemic and structural inequities to better serve survivors of marginalized identities [13].

Furthermore, our study documented the effects of COVID-19 on service providers and agency morale. The pandemic intensified role strain related to the personal (e.g., family, childcare, fear of illness) and professional demands for service providers. Macro-systems not only affect those who experience victimization, but also those who provide crisis services. Bolstering resilience efforts within the work setting is paramount in order to retain a healthy and engaged workforce [26]. Resilience building efforts must also acknowledge the intersectional identities of their advocates and consider the disproportionate effects that these macro-factors have on advocates who are also BIPOC. Despite these demands and challenges, providers’ remarked that their commitment to the mission of IPV advocacy, survivors, and to one another were sources of meaning, strength, and resilience.

There are several limitations to this study that must be acknowledged. First, our interviews were conducted with IPV service providers. However, we recognize that their sentiments may or may not be congruent with the lived experiences of survivors themselves, especially survivors who did not seek IPV-related services. Additionally, the study sample was recruited from IPV agencies in a medium-sized Midwestern city. Since the sample represents a small subset of IPV service providers, findings cannot be generalized, although they are still exploratory and idea-generating for IPV service providers in other locations. Additionally, the data were collected from June to August 2020, i.e., during the early phase of the pandemic. Therefore, our study was unable to explore the exacerbation of, and additional challenges raised by, the ongoing pandemic. Notwithstanding the limitations, this study is integral to shedding light on the issue of service providers’ thoughts on the impact of COVID-19 for IPV survivors and service provisions.

## 5. Conclusions

Our study highlights how COVID-19 sharpened a focus on the macro public health context of white privilege, racial inequity, and social and economic disparities. As eloquently stated by our participants, these themes share fertile ground with IPV vulnerability as they confer risk for exposure, more deleterious outcomes, and disparate access to resources for Black and Brown women [19]. Therefore, as next steps, our intent is to explore the ways in which these macro-contexts have shaped IPV services and whether there are gaps in culturally congruent services for Black and Brown women.

## Data Availability

Not applicable.
